# Unexpected Infective Endocarditis Presenting With Multiple Pulmonary Emboli Despite Negative High-Risk Imaging

**DOI:** 10.7759/cureus.102844

**Published:** 2026-02-02

**Authors:** Nyan Myint

**Affiliations:** 1 Internal Medicine, Meharry Medical College, Nashville, USA

**Keywords:** 2023 modified duke criteria, echo cardiogram, infective endocarditis, intravenous drug user endocarditis, staphylococcus aureus endocarditis

## Abstract

Infective endocarditis (IE) is notorious for causing septic emboli in major organs, especially the brain, spleen, and lungs. These emboli are expected to be caused by the shedding of vegetations that form on the endocardium of the heart. Therefore, positive findings of IE are generally expected on cardiac imaging, such as transthoracic echocardiography (TTE) and transesophageal echocardiography (TEE), when septic emboli are identified in a high-risk patient. This case highlights negative TTE and TEE findings in a patient with infective endocarditis who had an extensive burden of bilateral, multiple pulmonary emboli.

## Introduction

Right-sided infective endocarditis (IE) refers to IE involving the tricuspid and/or pulmonic valve. Intravenous drug users (IVDU) are at high risk for right-sided IE, accounting for approximately 90% of cases [1]. The possibility of right-sided IE should be suspected in an IVDU presenting with fever. The diagnosis can be confirmed using the 2023 Duke-International Society for Cardiovascular Infectious Diseases (2023 Duke-ISCVID) criteria. The initial evaluation of IE includes obtaining two sets of blood cultures and performing echocardiography, both of which constitute major diagnostic criteria (microbiologic and imaging) according to the 2023 Duke-ISCVID criteria. Transesophageal echocardiography (TEE) has higher sensitivity than transthoracic echocardiography (TTE) and is mandatory in patients with prosthetic valves, cardiac devices, or when there is high suspicion of IE complications, such as intracardiac fistula, paravalvular lesions, or perforation [2]. According to an external validation study, the 2023 Duke-ISCVID criteria demonstrated greater sensitivity and specificity compared to the modified Duke criteria. Microbiologic and imaging findings were the most significant contributors to the 2023 Duke-ISCVID criteria, being positive in 91% and 78.4% of definite IE cases, respectively. The removal of either criterion would result in more than a 50% reduction in the correct classification of definite IE [3]. Therefore, diagnosing IE can be challenging when one or both major criteria are negative, particularly when the imaging criterion is not met.

## Case presentation

A 55-year-old male presented with fatigue and generalized weakness for two months, and hematuria for one week. The generalized weakness was associated with a non-productive cough, night sweats, anorexia, and generalized joint pain. He denied fever with chills, orthopnea, paroxysmal nocturnal dyspnea, bilateral lower limb swelling, focal neurological deficits, skin rashes, or other skin changes. The hematuria was not associated with dysuria, urgency, urinary retention, or incontinence. Relevant past medical history included intravenous drug use (IVDU), with the last use reported two months prior, and gastroesophageal reflux disease. There was no known history of infective endocarditis (IE), valvular or congenital heart disease, cardiac surgery, or the presence of prosthetic heart valves or devices.

Physical examination was notable only for generalized weakness. Specifically, there were no murmurs, jugular venous distention, abdominal tenderness, renal angle tenderness, bilateral lower limb edema, Osler nodes, Janeway lesions, or Roth spots. Vital signs on presentation were as follows: blood pressure 118/75 mmHg, heart rate 130 beats per minute (bpm), respiratory rate 22 breaths per minute, temperature 98.7°F, oxygen saturation 98% on room air, body mass index 24.4 kg/m^2^. Urinalysis showed proteinuria (2+) and hematuria (3+), with 20 red blood cells per high-power field (HPF) on microscopic examination. Blood and serum laboratory results are shown in Table 1.

**Table 1:** Blood and serum laboratory test results, along with their reference ranges, at the time of presentation. eGFR: estimated glomerular filtration rate; BNP: brain natriuretic peptide; TSH: thyroid-stimulating hormone level; Free T4: free thyroxine level; CK: creatine kinase; ALP: alkaline phosphatase; AST: aspartate aminotransferase; ALT: alanine aminotransferase; MCV: mean corpuscular volume; WBC: white blood cells

Blood or serum tests	Results	Reference range
WBC (×10³ cells/µL)	20.9	4-11
Neutrophils (×10³ cells/µL)	18.17	1.5-7.5
Hemoglobin (g/dL)	11.8	13.5-17.5
Hematocrit (%)	34	40-54
MCV (fL)	76.6	80-100
Platelets (x10^9^/L)	217	150-400
Sodium (mmol/L)	128	135-145
Potassium (mmol/L)	4.3	3.5-5
Chloride (mmol/L)	84	96-106
Bicarbonate (mmol/L)	23	22-29
Glucose (mg/dL)	216	70-125
Calcium (mg/dL)	8.3	8.5-10.5
Urea (mg/dL)	56	7-20
Creatinine (mg/dL)	2.07	0.6-1.3
eGFR (mL/min/1.73 m²)	43	>60
Albumin (g/dL)	1.7	3.5-5.5
ALT (U/L)	74	10-49
AST (U/L)	162	8-48
ALP (U/L)	129	44-147
Total bilirubin (mg/dL)	1.9	0.1-1.2
Sedimentation rate (mm/h)	130	0-20
C-reactive protein (mg/L)	336.7	0-3.0
Lactic acid (mmol/L)	3.7	<2
BNP (pg/mL)	7436	<88
CK (U/L)	1820	41-331
TSH (µU/mL)	0.651	0.5-4
Free T4 (ng/dL)	1.1	0.8-1.8

**Figure 1:**
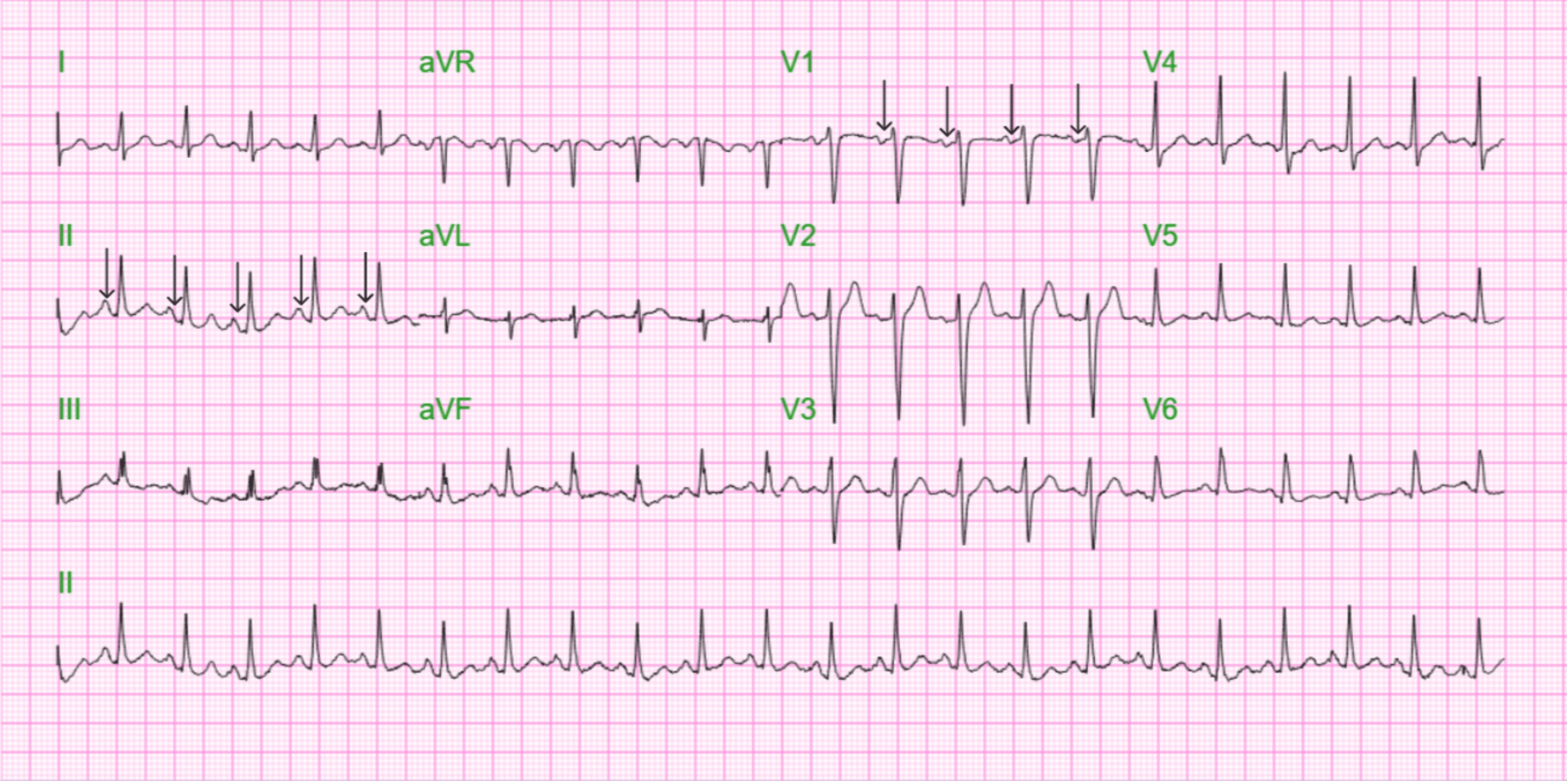
A 12-lead electrocardiogram (EKG) showing sinus tachycardia at 132 beats per minute, with no prolonged PR interval or axis deviation. The arrows indicate a normal PR interval in the PR segments in leads II and V1.

**Figure 2:**
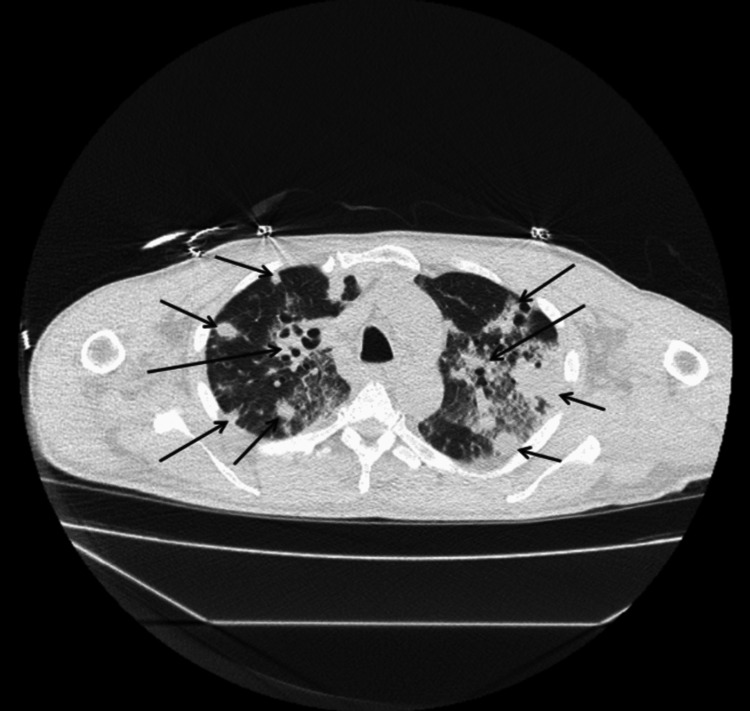
CT chest axial view without contrast showing upper parts of both lungs. The arrows show multiple, bilateral diffuse airspace cavitations with air bronchograms in some cavities, consistent with septic pulmonary emboli.

**Figure 3:**
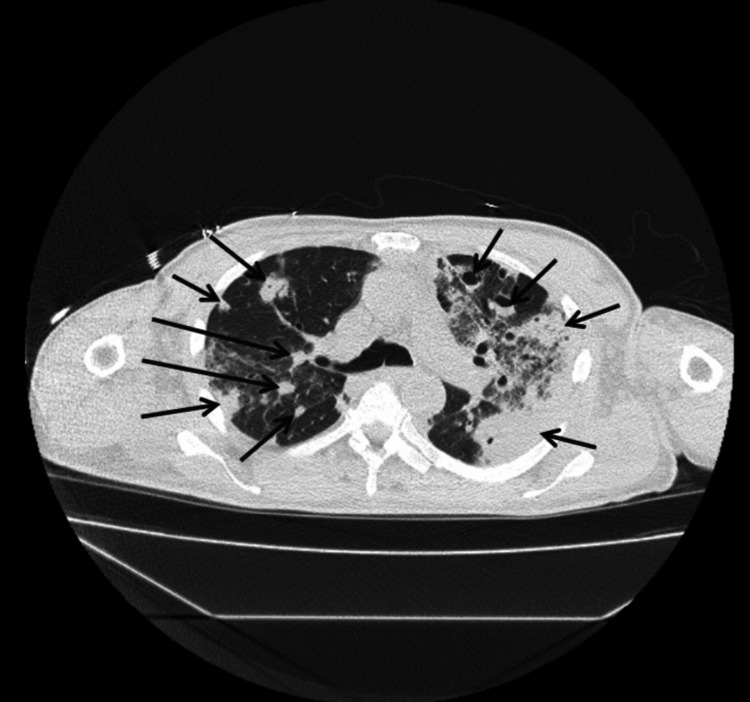
CT chest axial view without contrast showing upper-middle parts of both lungs. The arrows show multiple, bilateral diffuse airspace cavitations with air bronchograms in some cavities consistent with septic pulmonary emboli.

**Figure 4:**
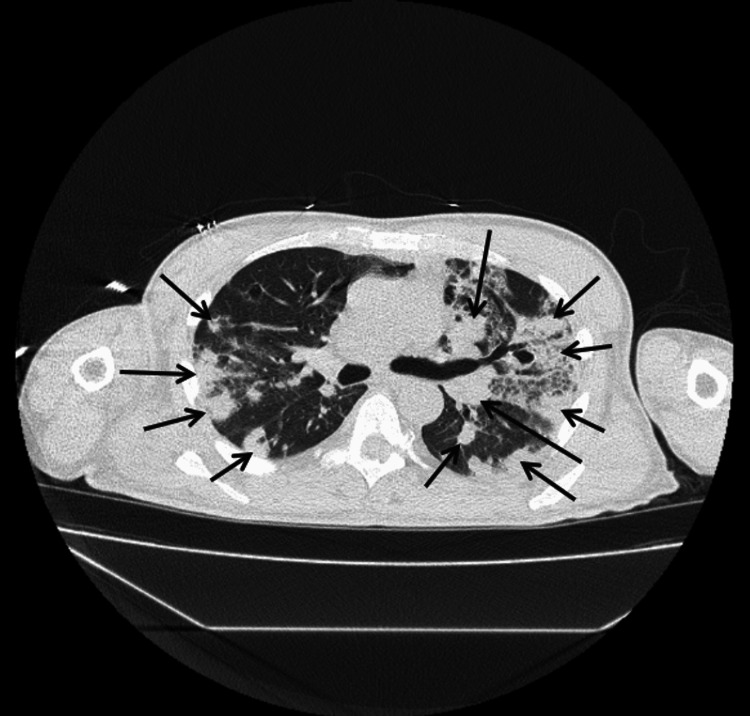
CT chest axial view without contrast showing the lower-middle parts of both lungs. The arrows show multiple, bilateral diffuse airspace cavitations with air bronchograms in some cavities, consistent with septic pulmonary emboli.

**Figure 5:**
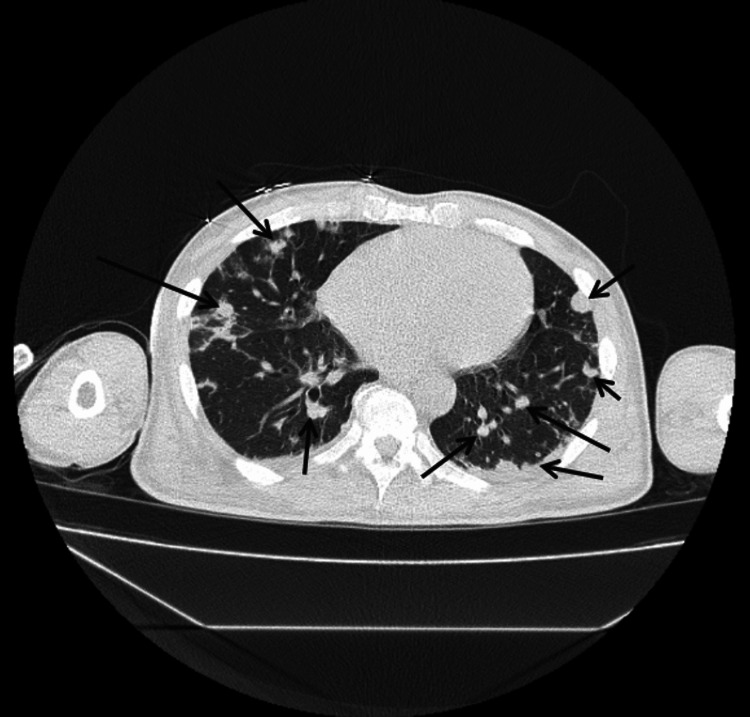
CT chest axial view without contrast showing the lower parts of both lungs. The arrows show multiple, bilateral diffuse airspace cavitations with air bronchograms in some cavities, consistent with septic pulmonary emboli.

**Figure 6:**
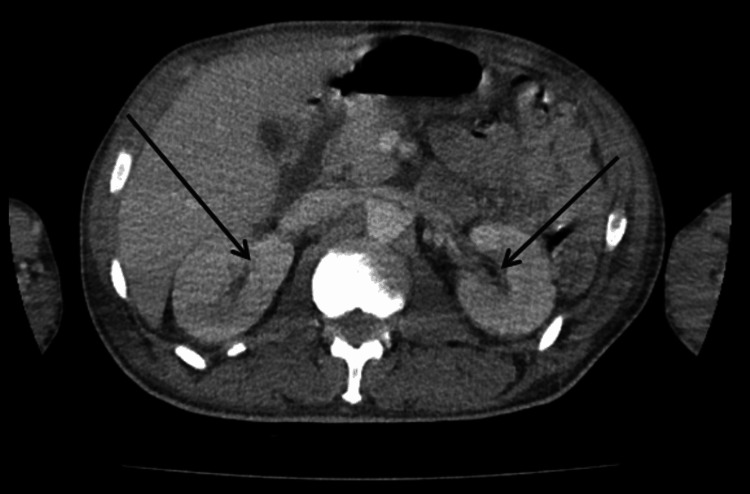
CT abdomen axial view without contrast showing middle part of the abdomen. The arrows are to illustrate that there was no hydronephrosis, perinephric fat stranding, or renal calculi.

Figure 1 illustrates 12-lead electrocardiogram (EKG) with sinus tachycardia at 132 bpm and no prolonged PR interval or axis deviation. A non-contrast computed tomography (CT) scan of the chest, abdomen, and pelvis was performed on the day of presentation as part of the diagnostic evaluation. Figures 2-5 show transverse chest CT images without contrast, revealing diffuse, bilateral alveolar airspace disease with cavitations consistent with septic emboli. Figure 6 shows a transverse CT image of the abdomen without contrast, demonstrating no hydronephrosis, perinephric fat stranding, or renal calculi.

**Figure 7:**
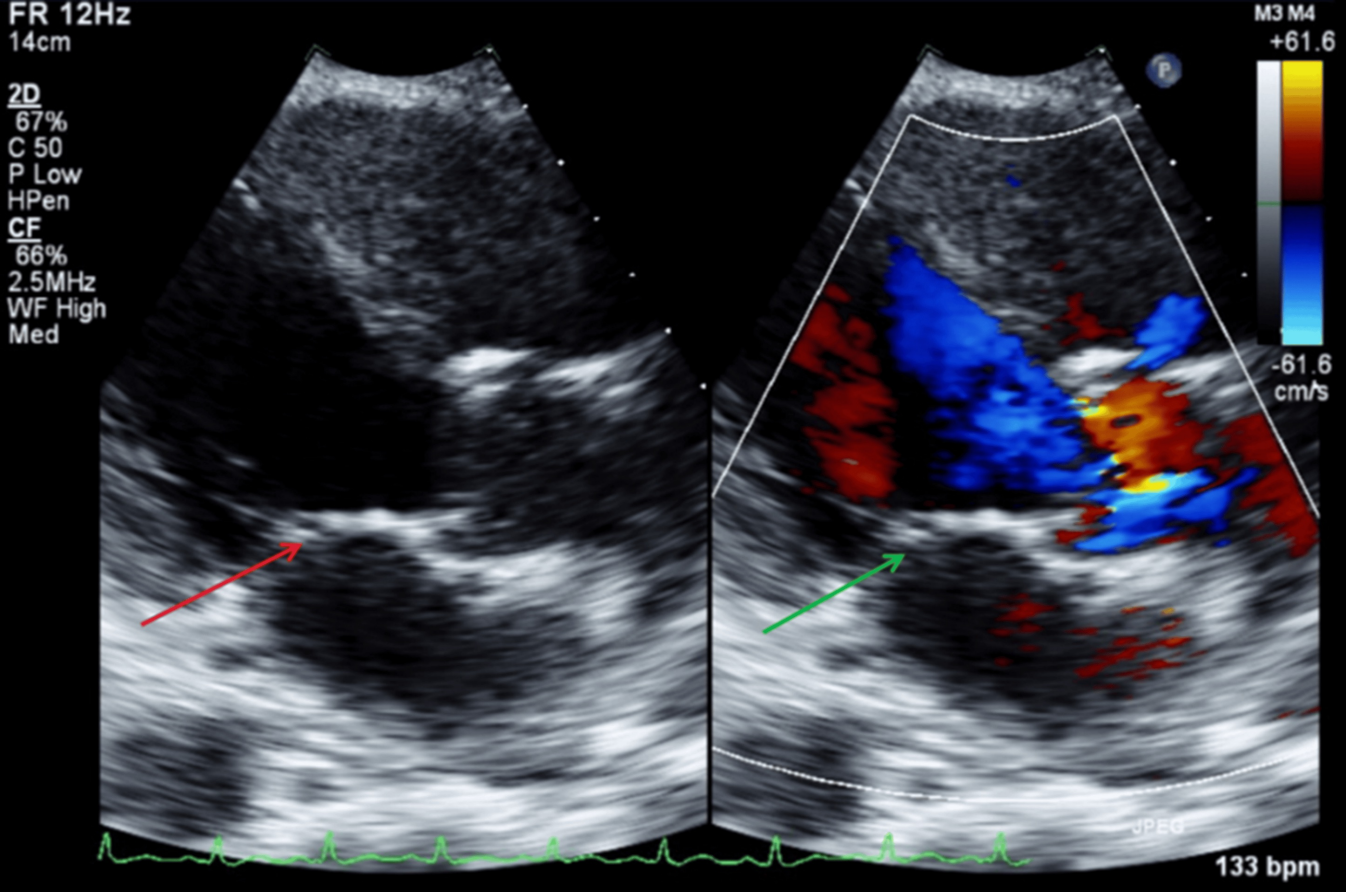
TTE parasternal long-axis view with and without color Doppler during systole. The red arrow shows mitral valve with no visible vegetation. The green arrow shows no findings of mitral valve regurgitation with color doppler. TTE: transthoracic echocardiography

**Figure 8:**
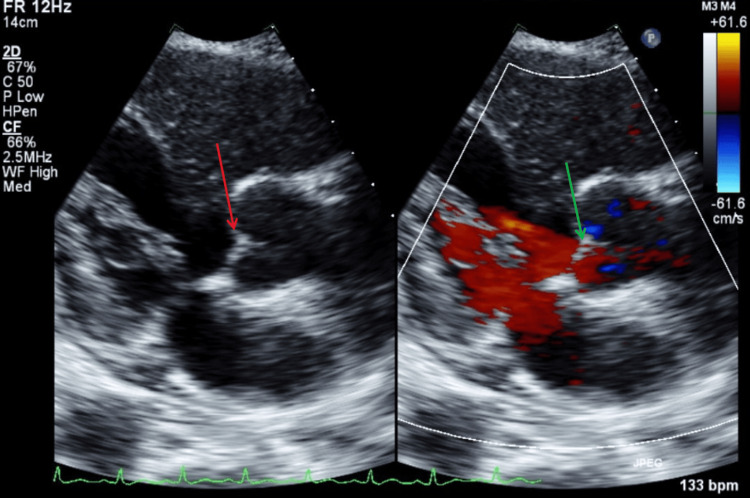
TTE parasternal long-axis view with and without color Doppler during diastole. The red arrow shows aortic valve without vegetations. The green arrow shows no signs of aortic valve regurgitation with color Doppler. TTE: transthoracic echocardiography

A transthoracic echocardiogram (TTE) was performed on the day of presentation in the setting of septic pulmonary emboli in an intravenous drug user (IVDU). No vegetations, abscesses, fistulas, or significant valvular regurgitation were identified on the TTE. Figure 7 illustrates a parasternal long-axis TTE view with and without color Doppler, showing no evidence of mitral regurgitation or vegetation during systole. Figure 8 illustrates a parasternal long-axis TTE view with and without color Doppler, showing no aortic regurgitation or vegetation during diastole.

**Figure 9:**
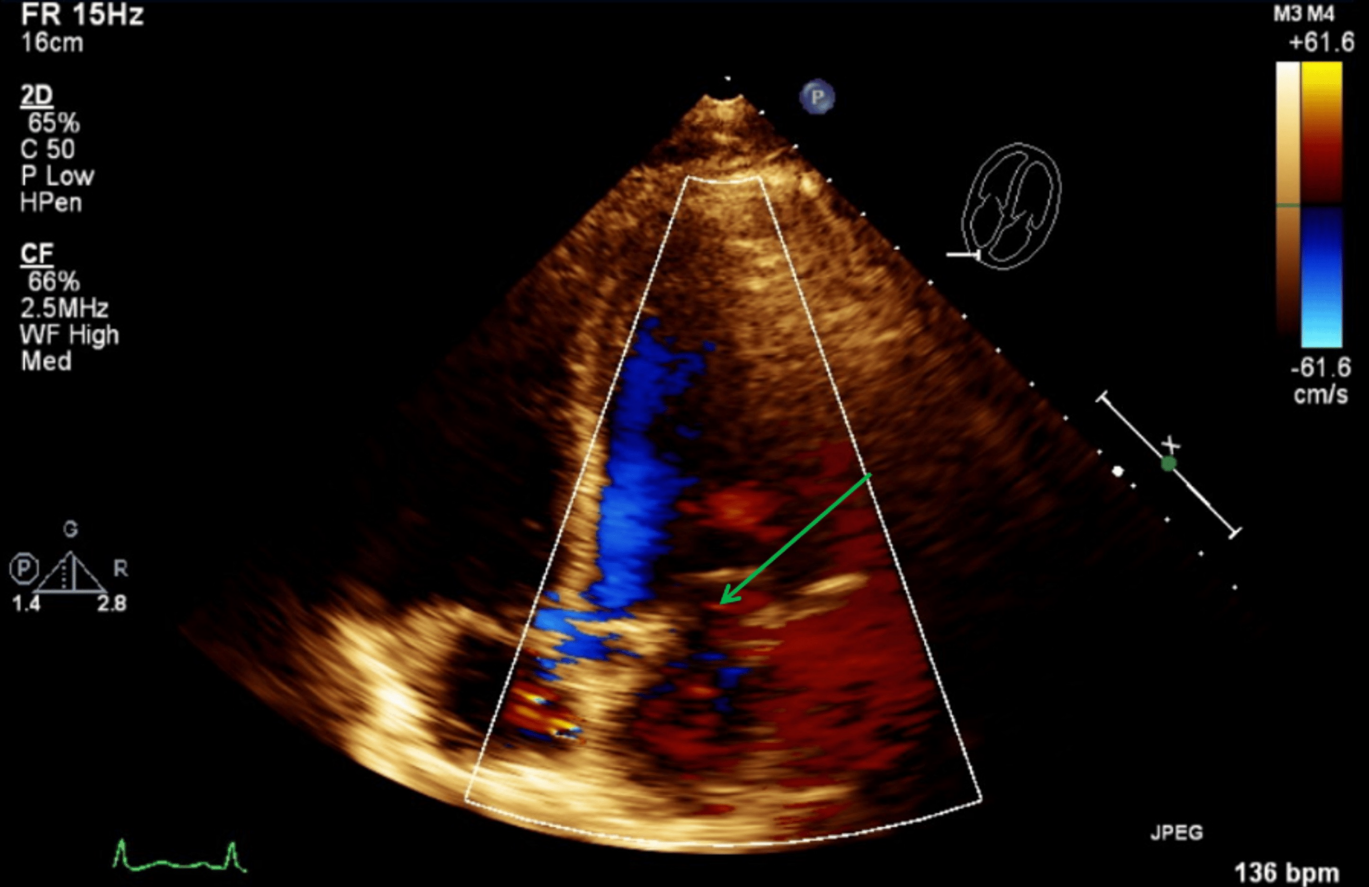
TTE apical three-chamber view with color Doppler during systole. The green arrow shows no findings of mitral regurgitation with color Doppler. TTE: transthoracic echocardiography

**Figure 10:**
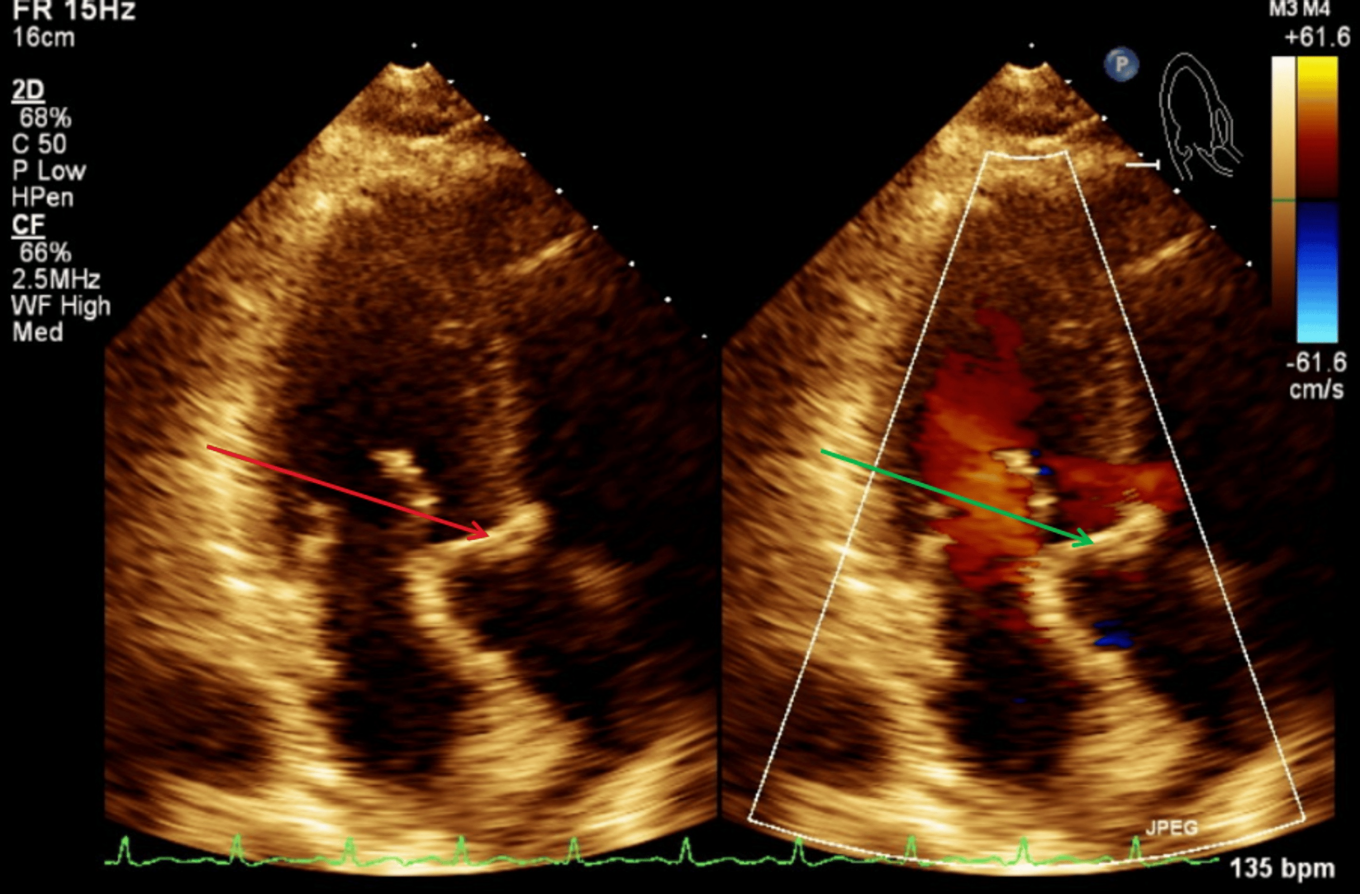
TTE apical three-chamber view with and without color Doppler during diastole. The red arrow shows aortic valve without visible vegetations. The green arrow shows no findings of aortic valve regurgitation with color Doppler. TTE: transthoracic echocardiography

Figure 9 illustrates a TTE apical three-chamber view with color Doppler, showing no mitral regurgitation or vegetations during systole. Figure 10 illustrates a TTE apical three-chamber view with and without color Doppler, showing no aortic regurgitation or vegetations during diastole.

**Figure 11:**
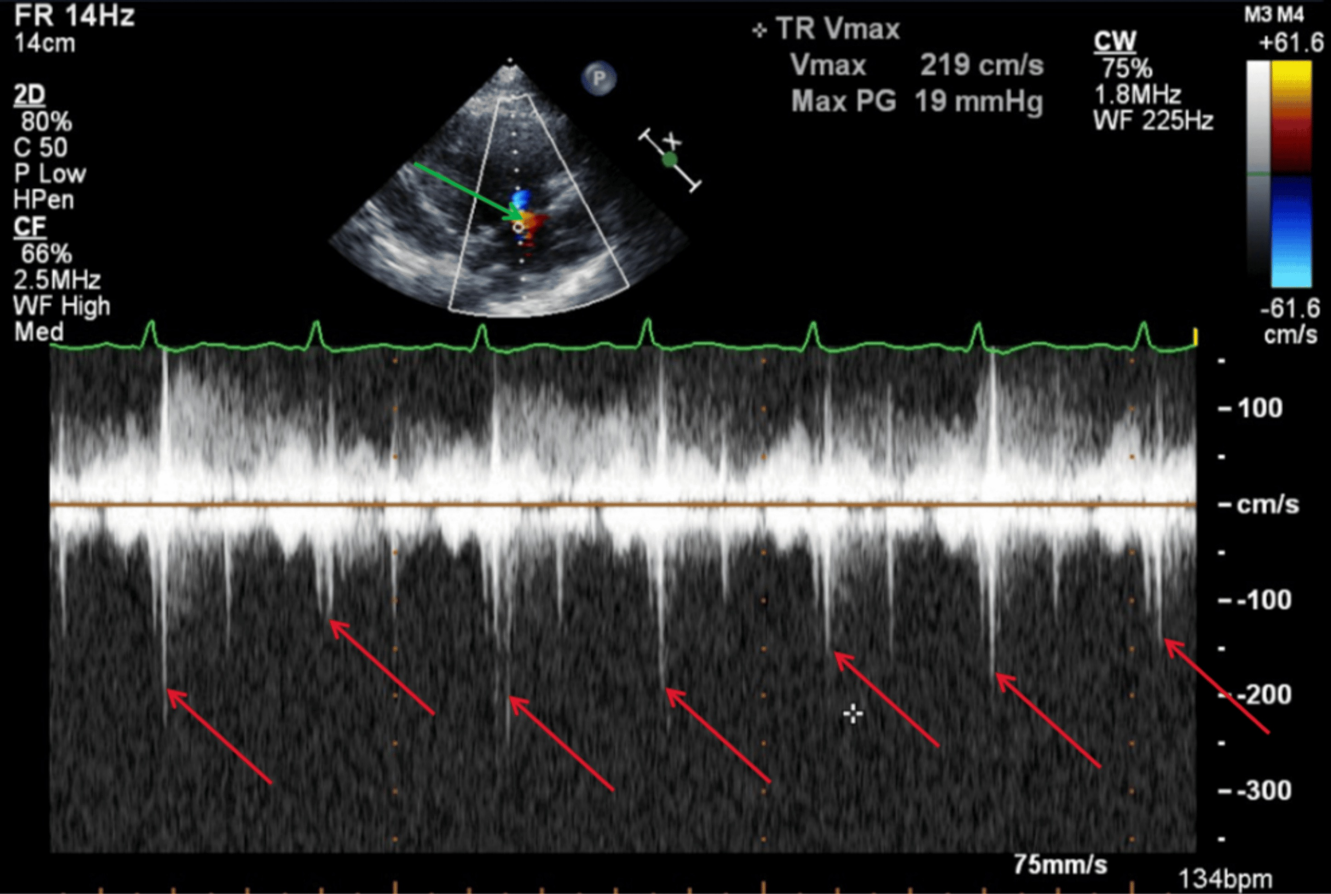
TTE parasternal short-axis view with color Doppler and M-mode during systole. The green arrow shows findings of mild tricuspid valve regurgitation with color Doppler. The red arrows show mild tricuspid valve regurgitation with various velocities with each systole. TTE: transthoracic echocardiography

**Figure 12:**
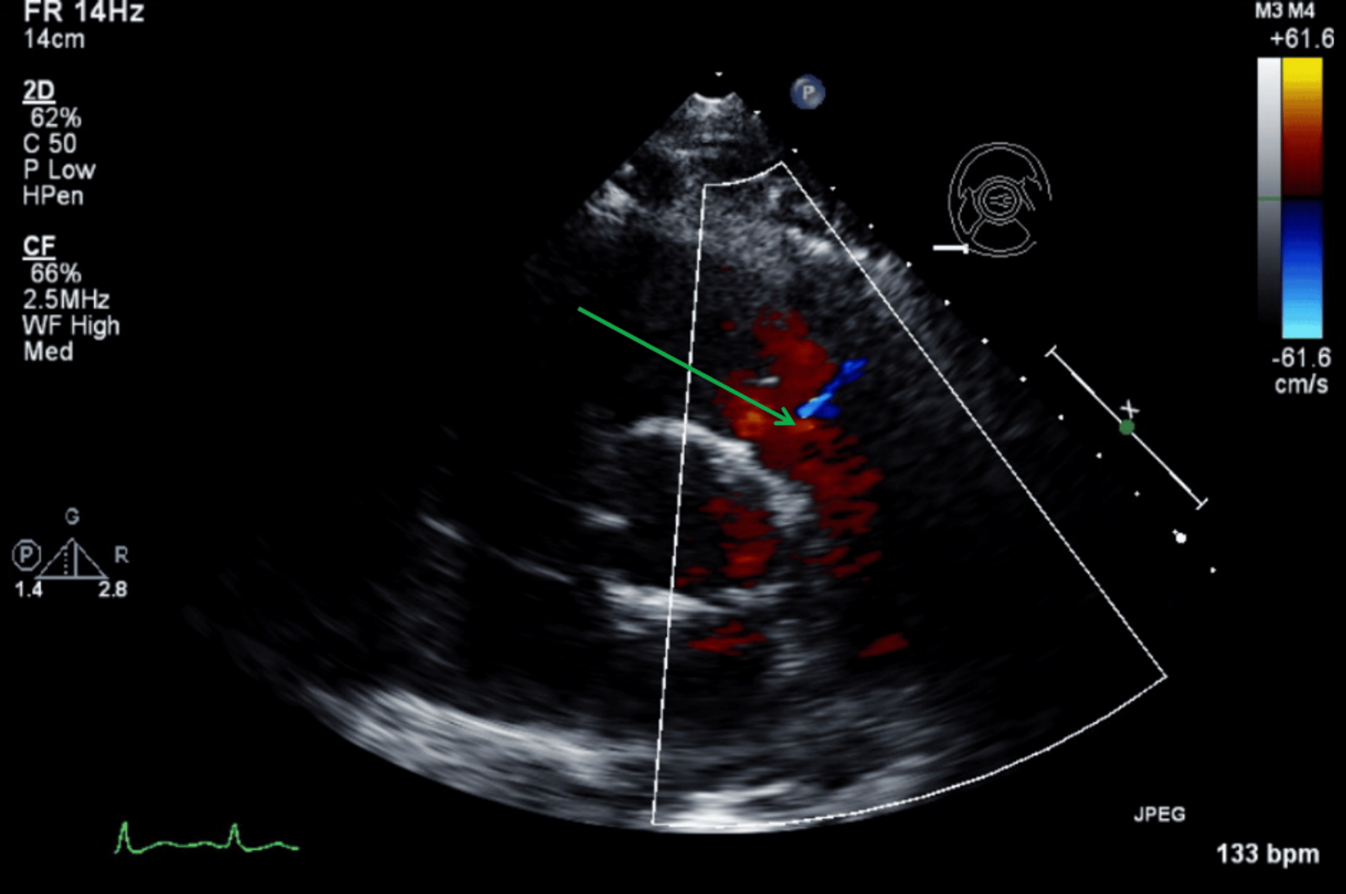
TTE parasternal short-axis view with color Doppler during diastole. The green arrow shows findings of mild pulmonic valve regurgitation with color Doppler. TTE: transthoracic echocardiography

Figure 11 illustrates a TTE parasternal short-axis view with color Doppler and M-mode, showing mild tricuspid regurgitation during systole. Figure 12 illustrates a TTE parasternal short-axis view with color Doppler, showing mild pulmonic valve regurgitation during diastole.

Two sets of blood cultures grew methicillin-resistant *Staphylococcus aureus* (MRSA) within 24 h of collection. The patient was started on intravenous (IV) vancomycin targeting a trough level of 15-20 µg/mL, and piperacillin-tazobactam 4.5 g every 6 h for broad-spectrum coverage in the setting of sepsis. Acute kidney injury improved with IV antibiotics and fluid resuscitation. A definite diagnosis of infective endocarditis (IE) was established according to the 2023 Duke-ISCVID criteria, with the following one major criterion (positive microbiologic evidence) and three minor criteria: predisposition (intravenous drug use), vascular phenomena (septic pulmonary emboli), and immunologic phenomena (immune complex-mediated glomerulonephritis, evidenced by acute kidney injury with hematuria and proteinuria). Other differentials, including tuberculosis and systemic fungal infection, were ruled out with three negative acid-fast bacilli sputum smears at least 8 h apart and negative serum and urine fungal antigen tests.

A multidisciplinary team, including infectious disease, cardiology, and cardiothoracic surgery, was involved in the patient’s care. Based on susceptibility testing, IV antibiotics were continued with vancomycin alone, targeting a trough level of 15-20 µg/mL. Blood cultures were repeated every 48 h until negative results were obtained. As the transthoracic echocardiogram (TTE) did not demonstrate positive imaging criteria, transesophageal echocardiography (TEE) was pursued to assess the potential need for cardiothoracic surgical intervention.

**Figure 13:**
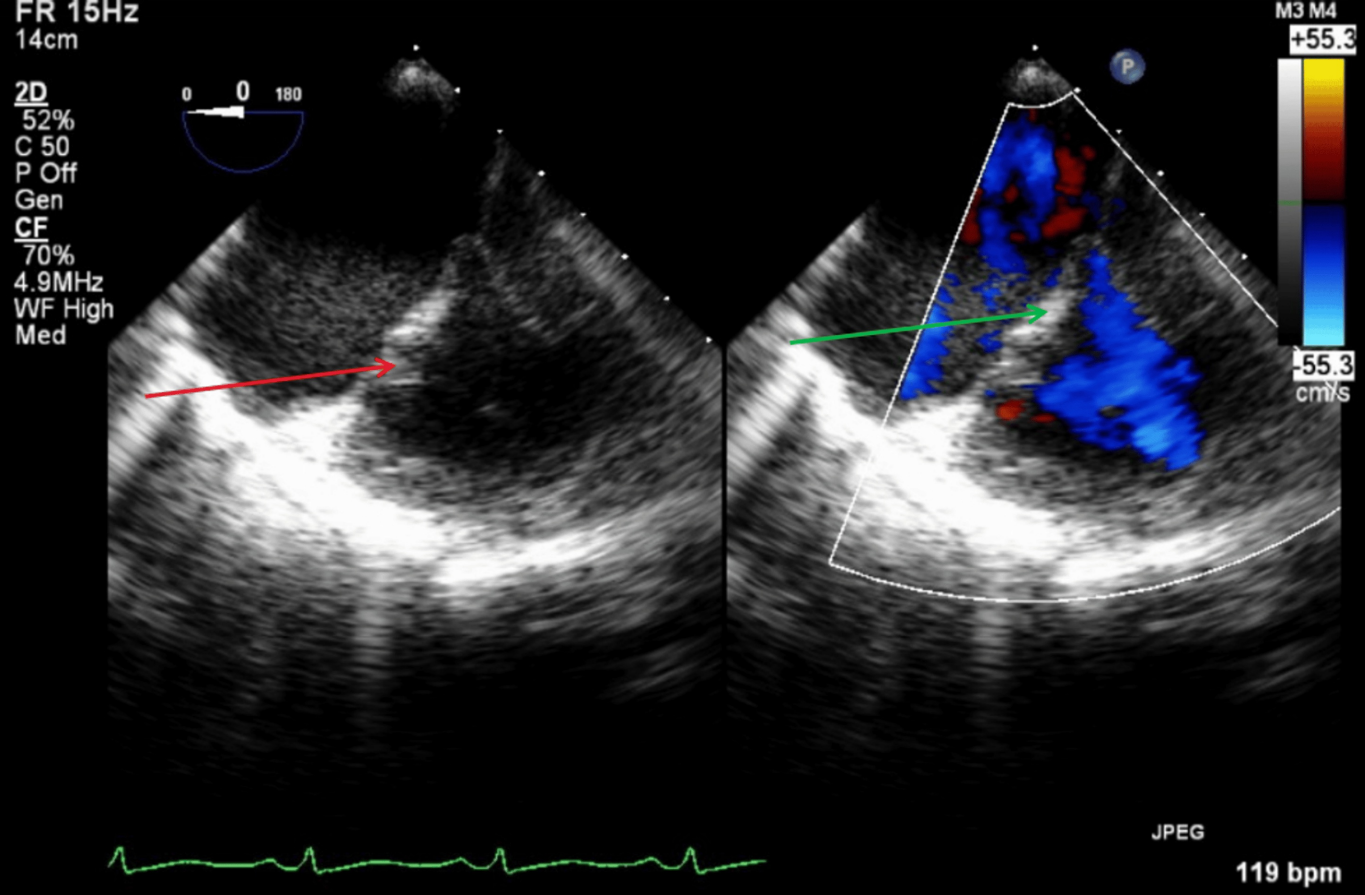
TEE mid-esophageal right two-chamber view with and without color Doppler during systole. The red arrow shows tricuspid valve with no visible vegetations. The green arrow shows no findings of tricuspid valve regurgitation with color Doppler. TEE: transesophageal echocardiography

**Figure 14:**
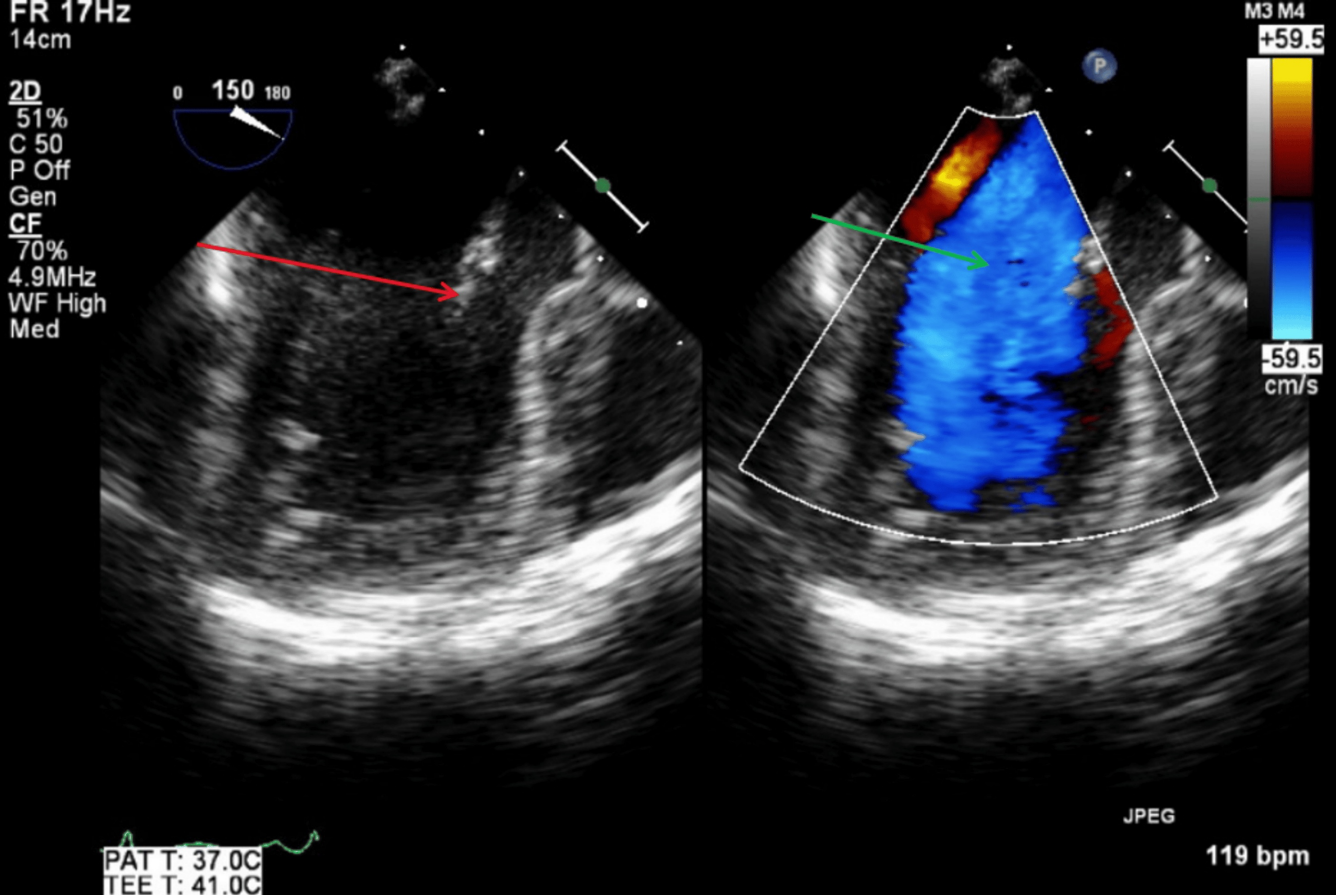
TEE mid-esophageal bi-commissural view with and without color Doppler during systole. The red arrow shows mitral valve with no visible vegetations. The green arrow shows no findings of mitral valve regurgitation with color Doppler. TEE: transesophageal echocardiography

TEE was performed on hospital day seven after the patient was stabilized, as persistent bacteremia had been present for more than seven days. TEE revealed no vegetations, abscesses, fistulas, or significant valvular regurgitation. Figure 13 illustrates a TEE mid-esophageal right two-chamber view with and without color Doppler, showing no tricuspid regurgitation or vegetations during systole. Figure 14 illustrates a TEE mid-esophageal bi-commissural view with and without color Doppler showing no mitral regurgitation or vegetations during systole.

**Figure 15:**
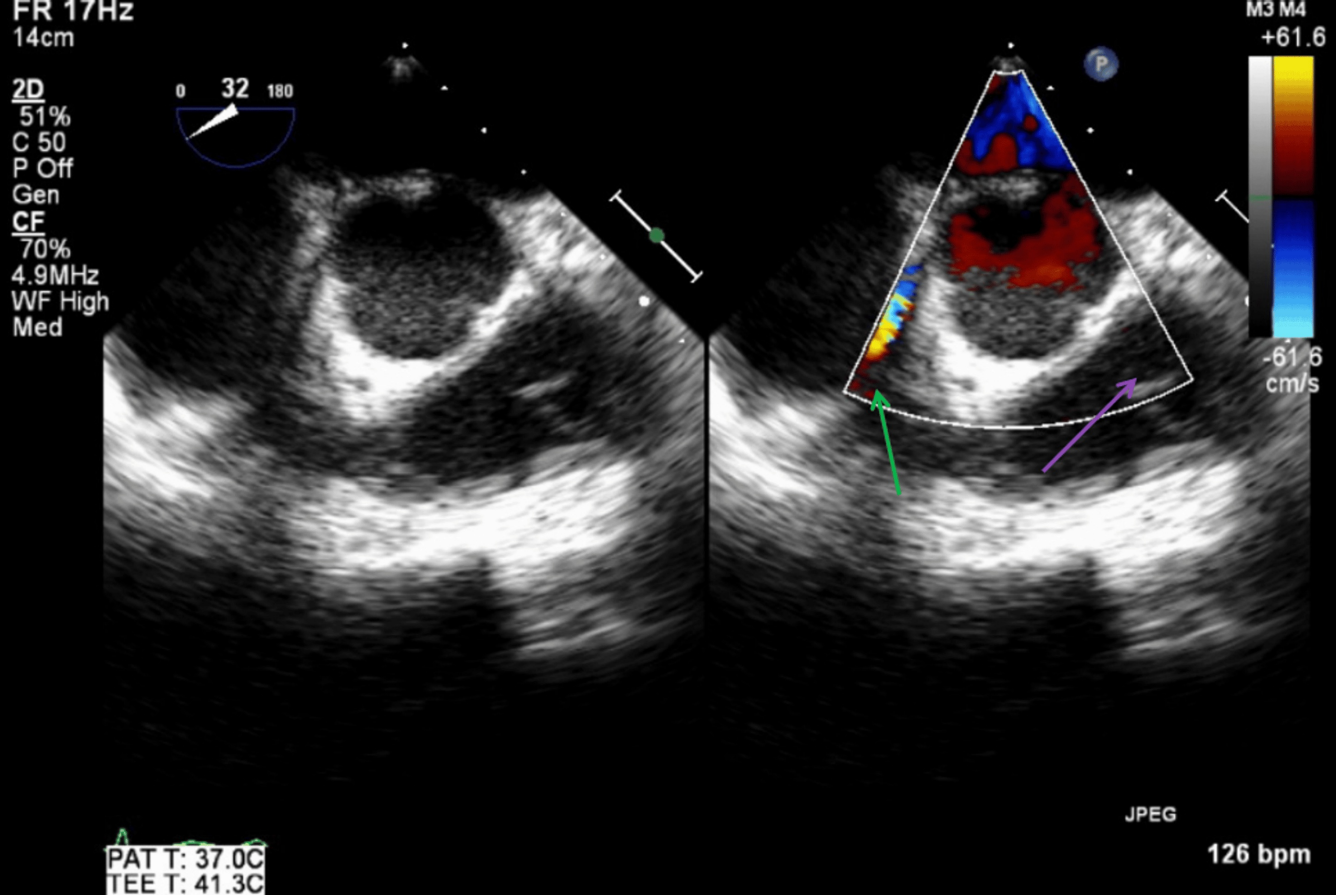
TEE mid-esophageal short-axis aortic valve view with and without color Doppler during diastole. The green arrow shows no findings of tricuspid valve regurgitation during diastole. The purple arrow shows no findings of pulmonic valve regurgitation during diastole. TEE: transesophageal echocardiography

**Figure 16:**
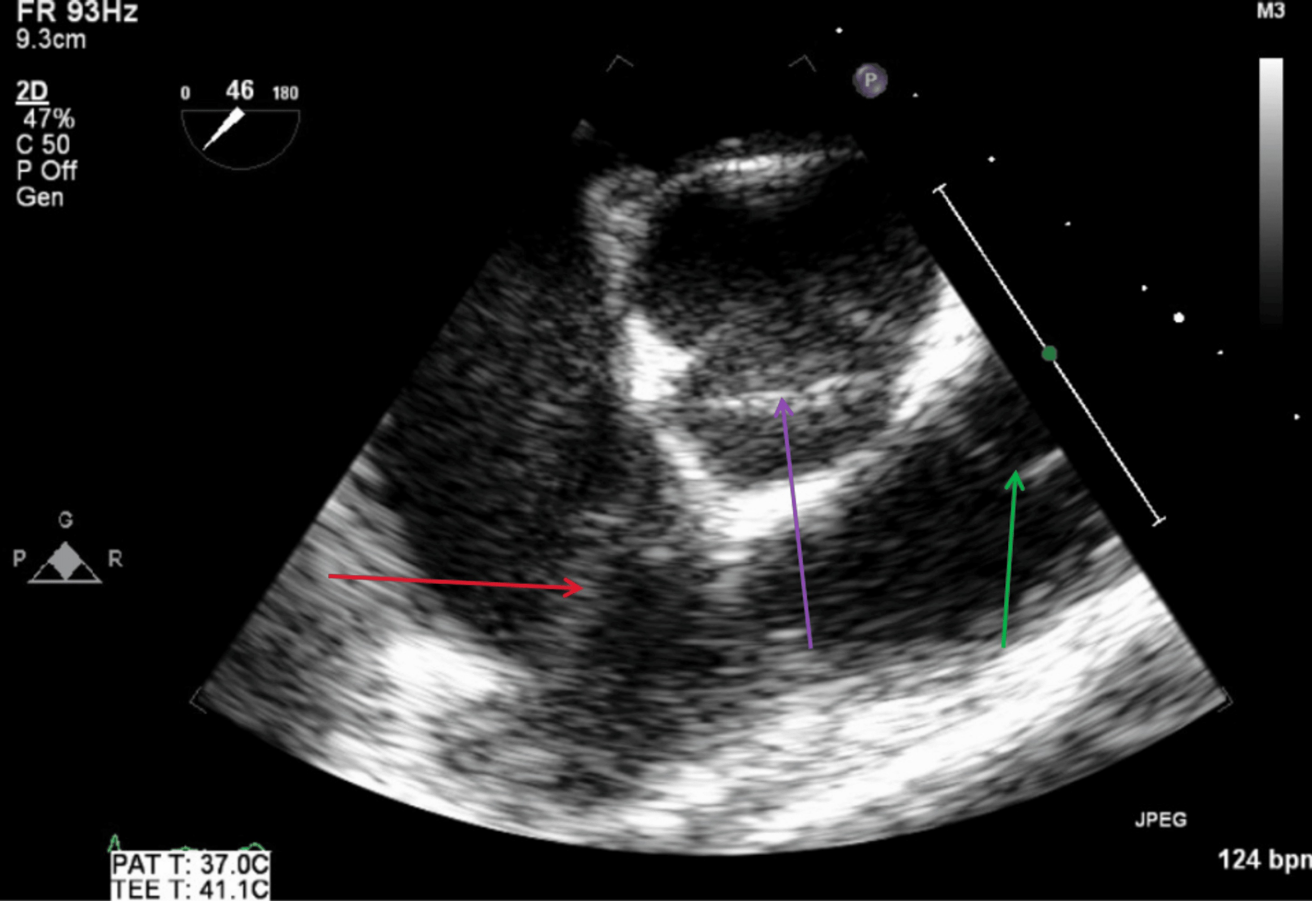
TEE mid-esophageal short-axis aortic valve view during diastole. The red arrow shows tricuspid valve with no visible vegetations. The purple arrow shows aortic valve with no visible vegetations. The green arrow shows pulmonic valve with no visible vegetations. TEE: transesophageal echocardiography

**Figure 17:**
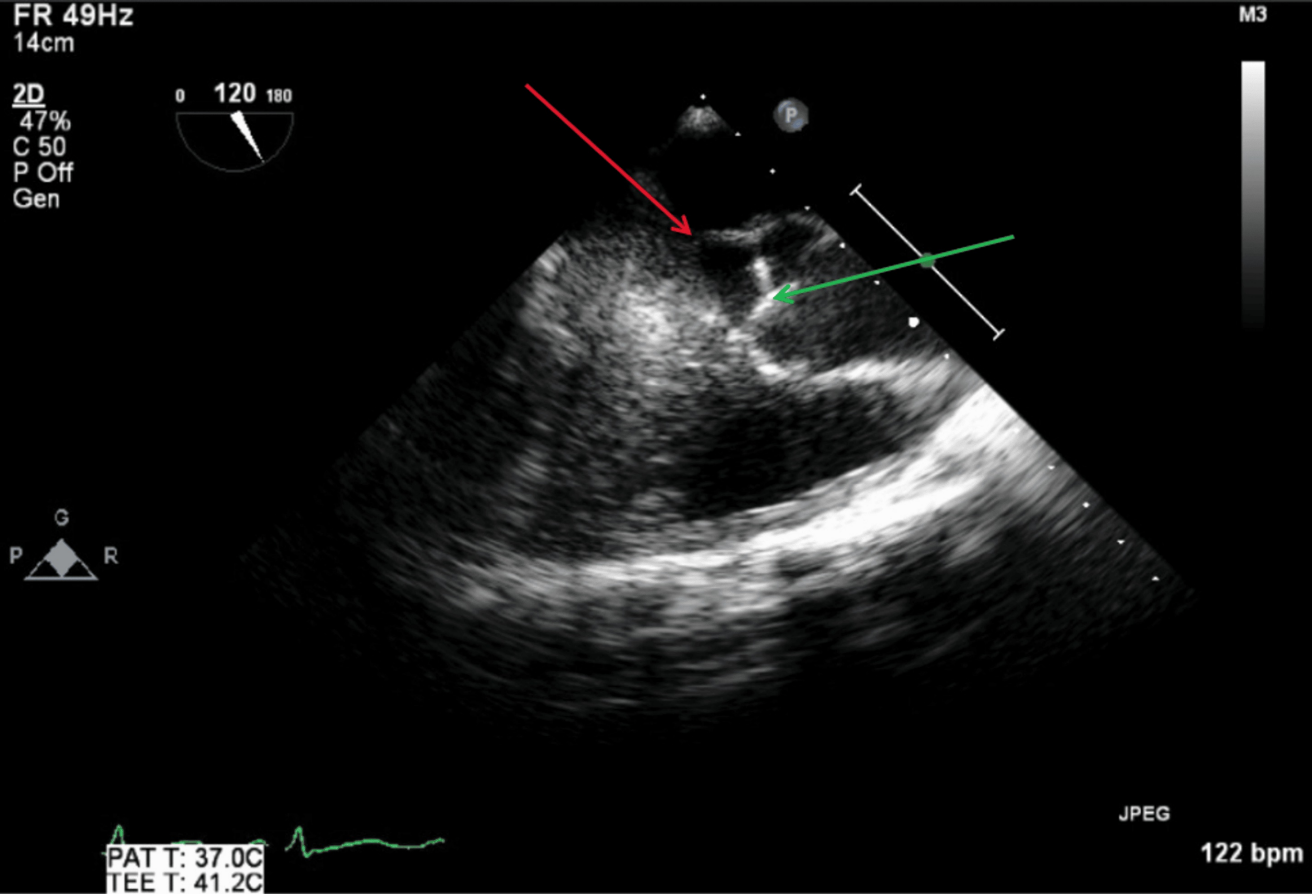
TEE mid-esophageal long-axis view during systole. The red arrow shows mitral valve with no visible vegetations. The green arrow shows aortic valve with no visible vegetations. TEE: transesophageal echocardiography

Figure 15 illustrates a TEE mid-esophageal short-axis aortic valve view with and without color Doppler, showing no aortic or pulmonic regurgitation during diastole. Figure 16 illustrates a TEE mid-esophageal short-axis aortic valve view, showing no abscesses, fistulas, or vegetations involving the tricuspid, aortic, or pulmonic valves. Figure 17 illustrates a TEE mid-esophageal long-axis view showing no abscesses, fistulas, or vegetations involving the mitral or aortic valves.

**Figure 18:**
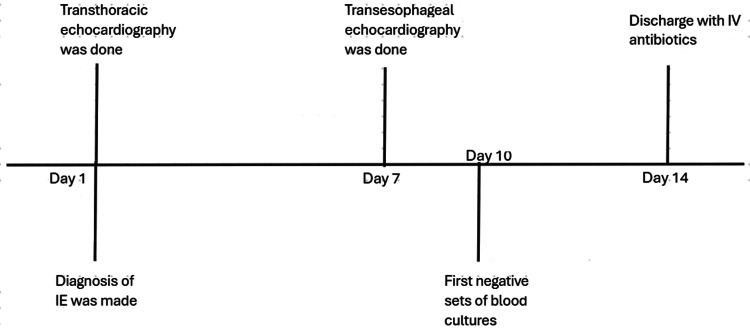
Case timeline to illustrating the timing of cardiac imaging and blood cultures with hospital days.

The potential need for surgical intervention due to persistent bacteremia, despite adequate and susceptible antibiotic therapy, was deemed unnecessary, as the TEE revealed no significant findings warranting surgery. The patient was managed conservatively with IV vancomycin under the guidance of the infectious disease team. The first set of blood cultures became negative on hospital day 10, which was confirmed by an additional set of blood cultures on day 12. The patient subsequently had a peripherally inserted central catheter (PICC) placed and was discharged home on hospital day 14. At discharge, the patient was prescribed an additional four weeks of home IV ceftaroline (total six weeks of antibiotics to prevent treatment failure rates with a shorter course in the setting of MRSA endocarditis), as recommended by the infectious disease team, for not requiring laboratory monitoring for either treatment side-effects or trough levels. Figure 18 is the case timeline summarizing the timeline of the imaging and blood cultures.

## Discussion

IVDU is more commonly associated with right-sided IE and is itself a predisposing factor, classified as one of the minor criteria in the 2023 Duke-ISCVID criteria. IVDU accounts for approximately one in every 10 invasive staphylococcal infections in the United States. *Staphylococcus aureus *is responsible for about 85% of right-sided IE cases among individuals who inject drugs [4]. A high index of suspicion for IE should be maintained in patients with IVDU, even when they present with non-specific or constitutional symptoms.

The 2023 Duke-ISCVID IE criteria can be used to establish a definite diagnosis of infective endocarditis (IE) if two major criteria, one major and three minor criteria, or five minor criteria are met. The 2023 Duke-ISCVID criteria demonstrate improved sensitivity compared to the 2000 modified Duke and 2015 European Society of Cardiology (ESC) criteria in patients with native valve, prosthetic valve, or cardiac implantable electronic device (CIED)-associated IE [5]. Therefore, we applied the 2023 Duke-ISCVID criteria to achieve a definite diagnosis of IE in patients with suspected IE. In this case, the patient met only one major criterion, the microbiologic major criterion, with two sets of blood cultures positive for methicillin-resistant *Staphylococcus aureus* (MRSA), while the imaging major criterion was negative. As previously mentioned, omission of the major imaging criterion could result in more than a 50% reduction in IE diagnoses according to the 2023 Duke-ISCVID criteria. The present case fulfilled three minor criteria, which compensated for the negative imaging major criterion.

Echocardiography is essential for both the diagnosis and management of IE. Findings such as new or significant valvular regurgitation, intracardiac masses or vegetations, fistulas, or abscesses constitute major imaging criteria for IE. In all IE suspected patients, TTE should be performed initially. TEE is recommended in patients with a high clinical suspicion for IE but negative or indeterminate TTE findings, as well as in those with high-risk features, such as new-onset heart failure, a new significant murmur, clinical stigmata of IE, a history of prior IE, or the presence of prosthetic valves or cardiac devices. TEE is also indicated when TTE reveals high-risk findings, such as large (>10 mm) or highly mobile vegetations. If clinical suspicion for IE persists despite an initial negative TEE, the study should be repeated within three to five days. TEE should also be repeated in patients with initially positive findings who subsequently experience clinical deterioration or fail to respond to antibiotic therapy. These recommendations align with the general principles outlined in the American Heart Association (AHA) guidelines for the diagnosis of IE in adults [6].

*Staphylococcus aureus* bacteremia is considered high risk for IE, and the VIRSTA score can be used to determine the need to pursue TEE after initial negative or indeterminate TTE. If the VIRSTA score is less than or equal to 2, TEE can be deferred, as the negative predictive value of the score is 98.8% compared to 17.4% with a VIRSTA score greater than 2 [7]. In this case, we decided to perform a TEE even after a definite IE diagnosis was established and no high-risk features were present, due to persistent bacteremia and a VIRSTA score of 16.

The sensitivity of TTE for diagnosing vegetations in native and prosthetic valves is 70% and 50%, respectively, while for TEE it is 96% and 92%, respectively. The specificity is around 90% for both TTE and TEE. Identification of vegetations may be difficult when they are small (<2-3 mm) or have already embolized. The sensitivity of TTE for diagnosing abscesses is about 50%, and for TEE is 90%. The specificity is greater than 90% for both TTE and TEE [8]. These data suggest that approximately 10% of vegetations or intracardiac abscesses can be missed using echocardiography. In the present case, the already embolized small vegetations were presumed to be the cause of the negative findings on both TTE and TEE.

Other imaging modalities that can be performed under the major imaging criteria include cardiac CT with multidetector CT (MDCT) and positron emission tomography CT imaging with fluorine-18 fluorodeoxyglucose (18F-FDG PET/CT). The accuracy of MDCT in detecting vegetation, abscess, and pseudoaneurysm is similar to that of TEE. The advantage of MDCT is that it provides better anatomic detail, which can be useful for surgical planning if needed. MDCT can also be used in patients with clinical indications of suspected pulmonary emboli. The major limitation of MDCT is the contraindication of iodinated contrast media in patients with renal dysfunction [9].

18F-FDG PET/CT has high accuracy for the diagnosis of IE in prosthetic heart valves and CIED, but its sensitivity is low in native valve IE, at 31%. Therefore, a negative 18F-FDG PET/CT cannot completely exclude native valve IE. An advantage of 18F-FDG PET/CT is its ability to identify septic emboli and distant lesions, except for cerebral septic emboli in the brain and intracerebral arteries. In this context, vascular phenomena can also be evaluated using this modality, as stated in the major imaging criteria [10]. A disadvantage is the potential risk of acute kidney failure from the contrast used in the study.

Diagnosis of IE without the imaging criterion, according to the 2023 Duke-ISCVID, can be made using other minor criteria, such as vascular and immunologic phenomena. Embolic events are common in left-sided IE and often occur early in the course of the disease. The incidence of embolic events in IE ranges from 20% to 50%. In a multicenter study on IE, embolic events were distributed as follows: 38% to the central nervous system (CNS), 30% to the spleen, 13% to the kidneys, 10% to the lungs, 6% to peripheral arteries, 2% to the mesentery, and 1% to the coronary arteries [11].

Right-sided IE is more frequently associated with septic pulmonary embolism, while cerebral and splenic septic embolisms are more common in left-sided IE. Embolic events may be clinically silent in about 20% of embolic IE cases. Embolism can occur before the diagnosis of IE is established, and the risk is highest during the first two weeks of antibiotic therapy. The highest risk is associated with large vegetations (>10 mm) and highly mobile vegetations. Other high-risk features include vegetations located on the anterior mitral leaflet of the mitral valve and *Staphylococcus aureus* IE [12]. Currently, there are no guidelines recommending routine screening for embolic events in IE, but surgical intervention is recommended for patients with the highest-risk features (vegetations ≥10 mm), particularly following an embolic event, to prevent further embolic complications.

In a study of 441 patients with left-sided IE, 190 experienced embolic events. Among these, 142 patients had vegetations, with 90 patients having vegetations ≥10 mm. Approximately 25% of patients with embolic events were diagnosed without any vegetations detected, even though TTE and TEE were used in the majority (≥80%) of patients [13]. These data suggest that embolic events can occur without any high-risk imaging findings, as was the case in our patient.

The patient was assumed to have right-sided tricuspid IE, as he was an IVDU presenting with multiple septic pulmonary emboli. Unlike left-sided valvular vegetations, tricuspid vegetations tend to be larger and are generally more easily visualized with echocardiography, with no significant difference between TTE and TEE [14]. Therefore, this case is unique in that there were no positive findings of IE on either TTE or TEE, despite the presence of multiple septic pulmonary emboli in a right-sided IE in an IVDU. We hypothesized that the vegetations may have fragmented and embolized, leaving only very small remnants or none in situ, which would render TTE and TEE ineffective.

This case may add incremental value to the existing literature on embolic events in infective endocarditis without detectable vegetations, particularly septic pulmonary emboli associated with right-sided infective endocarditis.

Limitations

Although the TEE was negative in this case, it was done after a week of negative TTE results and treatment, which could potentially affect the findings in the study. Follow-up imaging studies after discharge, such as chest CT and echocardiographic data, were unavailable due to loss of follow-up.

## Conclusions

Embolic events in IE can occur even without evidence of a positive imaging major criterion per the 2023 Duke-ISCVID criteria, although embolic events in IE are known to be associated with large vegetations. Proper utilization of the 2023 Duke-ISCVID criteria with diagnostic vigilance is important in the management of IE.
